# Learning in the Digital Era – Awareness and Usage of Free Open Access Meducation among Emergency Department Doctors

**DOI:** 10.7759/cureus.6223

**Published:** 2019-11-24

**Authors:** Sashriqua Palliam, Zeyn Mahomed, Deidre Hoffman, Abdullah E Laher

**Affiliations:** 1 Emergency Medicine, University of the Witwatersrand, Johannesburg, ZAF

**Keywords:** foam, free open access meducation, free open access medical education, digital medicine, emergency medicine, medical education

## Abstract

Introduction

Information and communication technology has revolutionized the space of medical education by providing a multitude of up-to-date evidence-based data to healthcare practitioners. Despite the increasing popularity of FOAM - Free Open Access Meducation (Medical Education) globally - data relating to its awareness and usage in Africa is lacking. In this study, we explore the awareness and usage of FOAM among doctors working at select emergency departments in Johannesburg.

Methods

The study comprised a prospective, questionnaire based, cross-sectional survey of medical doctors working at five academically affiliated emergency departments in Johannesburg. Data was described and compared.

Results

One-hundred and four participants completed the survey. Most of the respondents were aged between 31 and 39 years (n = 40, 43.9%). There were no significant differences between the proportion of females and males that used FOAM (p = 0.56). Most participants (n = 91, 87.5%) were aware of FOAM, while 82 (78.8%) used FOAM, 13 (12.5%) were unsure if they used FOAM and nine (8.7%) did not use FOAM. Majority of those that used FOAM, only used it once a week (n = 47, 57.3%). Most participants spent between one and two hours per day on FOAM (n = 29, 35.4%). Smartphones were by far the most commonly used device to access FOAM (n = 91, 87.5%).

Conclusion

The level of awareness of FOAM is high and its usage is prevalent among emergency medicine healthcare professionals in Johannesburg. As technology becomes more prominent, institutions must aim to adapt to the digital era in their teaching methods.

## Introduction

“If you want to know how we practiced medicine 5 years ago, read a textbook. If you want to know how we practiced medicine 2 years ago, read a journal. If you want to know how we practice medicine now, go to a good conference. If you want to know how we will practice medicine in the future, listen in the hallways and use free online medical education” [1].

The hallmark of the medical profession is to deliver health care that is consistent with patients’ needs within the modern health system [2]. Health care professionals are expected to stay abreast with recent medical developments, evolving health care standards and best clinical practice [3]. Information and communication technology has revolutionized the space of medical education [4]. Technologically agile pedagogical approaches that include the use of FOAM - Free Open Access Meducation (Medical Education) blogs - have dramatically advanced accessibility to continuous medical education globally [5].

FOAM originated in 2012 at the International Conference of Emergency Medicine in Dublin as a globally sourced and accessible educational resource to enhance traditional educational methodologies [6]. Over the years, there has been an increase in the number of Emergency Medicine and Critical Care blog databases with there being currently over 460 FOAM resources available [7].

FOAM provides an important medium for the sharing of ideas and fast-tracking the translation of medical developments into clinical practice [8]. It utilizes multiple online platforms such as podcasts, blogs, videos, tweets (Twitter), Facebook groups and other web-based media to develop and distribute medical education. These interactive databases have created a dynamic online community and a family of FOAM users worldwide [9]. Since FOAM leans towards self-directed problem-based learning, various medical schools across the world have also embedded it into their curriculum [10-12].

Despite the increasing popularity of FOAM globally, data relating to its awareness and usage in Africa is lacking. We therefore aimed to explore the awareness and usage of FOAM among doctors working at select emergency departments in Johannesburg.

## Materials and methods

This researcher-administered, questionnaire based, cross sectional study was conducted between 01 November 2017 and 31 January 2018. The study population comprised a convenience sample of 104 medical doctors working at the EDs of one of five hospitals in Johannesburg. All five hospitals were affiliated to the University of the Witwatersrand.

Questionnaires were distributed to potential participants (doctors) working at the respective EDs. Participants included emergency medicine physicians (EMPs), emergency medicine registrars (EMRs) and emergency medicine medical officers (MOs). Doctors registered to practice as specialists in the field of emergency medicine were classified as EMPs, whereas doctors that were training to become specialist in the field of emergency medicine were classified as EMRs. All other doctors who had completed their medical internship and were employed at each of the included hospitals EDs were classified as MOs. Medical interns and undergraduate students were excluded from the study.

After conducting a pilot study on five random subjects in July 2017, the final questionnaire was adapted from the questionnaire that was utilized in the study by Thurtle et al. [13]. The questionnaire assessed awareness and usage of FOAM, reasons for not using FOAM and devices used to access FOAM. In addition, the most popular FOAM blog used by study participants was also determined [8].

Ethical clearance to conduct the study was obtained from the Human Research Ethics Committee (Medical) of the University of the Witwatersrand (clearance certificate M170723). Further written approval was granted by the Chief Executive Officers and Heads of the Emergency Department of each of the participating hospitals.

The participant information sheet and questionnaire were distributed by the primary investigator to consenting participants at the respective departmental academic meetings. No identifying data was collected. The questionnaires were completed in confidence and returned into a sealed box that was provided.

Collected data was captured into an electronic data spread sheet (Microsoft® Excel®) and thereafter analyzed. Results were predominantly described using frequency and percentage tables and a graph. The Chi Square test was used to compare differences between the proportion of females and males that used FOAM blogs, while the Fisher's Exact test was used to compare the awareness and usage of FOAM between MOs, EMRs and EMPs. The level of significance was set at α = 0.05.

## Results

Of the 129 potential subjects that were approached, 104 completed the survey, giving a response rate of 80.6%. Fifty-one percent (n = 53) of respondents were female. Of the total number of respondents, MOs comprised 56.8% (n = 59), EMRs 28.8% (n = 30) and EMPs 14.4% (n = 15). Most of the respondents were aged between 31 and 39 years (n = 40, 43.9%). All the EMRs were under the age of 50 years, while all the EMPs were older than 30 years.

Most participants (n = 91, 87.5%) were aware of the existence of FOAM, however only one participant had started his own FOAM blog. Eighty-two (78.8%) participants used FOAM while 13 (12.5%) were unsure if they used FOAM and nine (8.7%) did not use FOAM. Majority of those that used FOAM, only used it once a week (n = 47, 57.3%). There were no significant differences between the proportion of females (n = 43) and males (n = 39) that used FOAM (p = 0.56). Smartphones were by far the most commonly used device to access FOAM (n = 91, 87.5%). Details of the above are described in Table 1.

**Table 1:** Description of gender, age group, awareness of FOAM, usage of FOAM, frequency of usage of FOAM and devices used to access FOAM among participants with various levels of training MO: Medical Officers; EMR: Emergency Medicine Registrars; EMP: Emergency Medicine Physicians; N/A: Not applicable.

	MO (n, %)	EMR (n, %)	EMP (n, %)
GENDER			
Female (n = 53, 51.0%)	32 (60.4)	10 (18.9)	11 (20.7)
Male (n = 51, 49.0%)	27 (52.9)	20 (39.2)	4 (7.9)
AGE GROUP (YEARS)			
≤30 (n = 42, 40.4%)	34 (80.9)	8 (19.1)	0 (0)
31–39 (n = 44, 42.3%)	15 (34.1)	19 (43.2)	10 (22.7)
40–49 (n = 11, 10.6%)	6 (54.6)	3 (27.2)	2 (18.2)
≥50 (n = 7, 6.7%)	4 (57.1)	0 (0)	3 (42.9)
AWARENESS OF FOAM			
Entire cohort (n = 91, 87.5%)	47 (51.6)	29 (31.9)	15 (16.50)
≤30 years (n = 37, 40.7%)	29 (78.4)	8 (21.6)	N/A
31–39 years (n = 40, 43.9%)	12 (30.0)	18 (45.0)	10 (25.0)
40–49 years (n = 10, 11.0%)	5 (50.0)	3 (30.0)	2 (20.0)
≥50 years (n = 4, 4.4%)	1 (25.0)	N/A	3 (75.0)
USAGE OF FOAM			
Entire cohort (n = 82, 78.8%)	41 (50.0)	28 (34.1)	13 (15.9)
≤30 years (n = 31, 37.8%)	24 (77.4)	7 (22.6)	N/A
31–39 years (n = 39, 47.6%)	12 (30.8)	18 (46.1)	9 (23.1)
40–49 years (n = 8, 9.8%)	4 (50.0)	3 (37.5)	1 (12.5)
≥50 years (n = 4, 4.8%)	1 (25.0)	N/A	3 (75.0)
FREQUENCY OF USAGE OF FOAM			
Daily (n = 24, 29.3%)	9 (37.5)	10 (41.7)	5 (20.8)
Weekly (n = 47, 57.3%)	24 (51.0)	17 (36.2)	6 (12.8)
Monthly (n = 11, 13.4%)	8 (72.7)	1 (9.1)	2 (18.2)
DEVICES USED TO ACCESS FOAM			
Smartphone (n = 91, 87.5%)	50 (54.9)	28 (30.8)	13 (14.3)
Tablet (n = 37, 35.6%)	12 (32.4)	15 (40.5)	10 (27.1)
Computer/Laptop (n = 49, 47.1%)	22 (44.9)	17 (34.7)	10 (20.4)
MP3/iPOD (n = 2, 1.9%)	1 (50.0)	1 (50.0)	0 (0)

**Figure 1:**
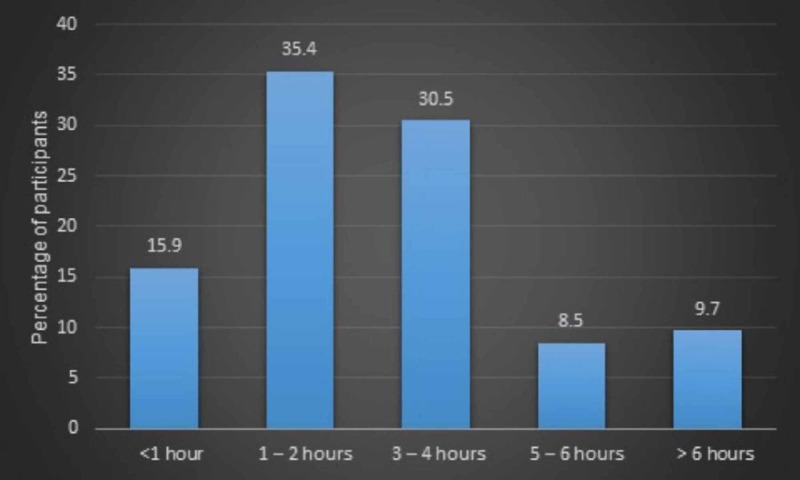
Hours spent per day on FOAM

Awareness and usage of FOAM was significantly lower in MOs compared to EMRs (p = 0.024 and p = 0.014, respectively). There were no statistically significant differences between MOs and EPs (p = 0.109 and p = 0.327, respectively) or EMRs and EMPs (p = 1.00 and p = 0.591, respectively). Figure 1 describes the number of hours spent by participants per day on FOAM. Most participants spent between one and two hours per day on FOAM (n = 29, 35.4%).

Among the nine respondents that did not use FOAM, reasons included: unfamiliarity with social media (n = 4, 44.4%); lack of time (n = 3, 33.4%); lack of access to free internet in the department (n = 1, 11.1%) and reluctance to use social media (n = 1, 11.1%). Most of the participants (n = 83, 79.9%) reported that a tutorial on how to access FOAM would increase their use of FOAM. More than three-quarter of subjects (n = 82, 78.8%) agreed that FOAM is an interactive tool, while 74 (71.2%) agreed that FOAM encouraged collaboration within the medical community. The most popular FOAM blog among participants is described in Table 2.

**Table 2:** Most popular FOAM blog among participants

FOAM blog	Country of Origin	n (%)
Life in the Fast Lane	Australia	67 (73.6)
EmCrit	USA	8 (8.8)
Academic Life in Emergency Medicine	USA	6 (6.6)
FOAM EM RSS	USA	4 (4.4)
St Emlyn’s	England	2 (2.2)
The Skeptics Guide to Emergency Medicine	Canada	2 (2.2)
Pulm CCM	USA	1 (1.1)
Resus.me	USA	1 (1.1)
Boring EM	Canada	0 (0)
The Trauma Professional’s Blog	USA	0 (0)

## Discussion

It is rare to find a profession that has not been impacted by the digital revolution. The ubiquity and rapid increase in availability of digital media in the field of emergency medicine has led to calls for the formal integration of online learning into residency training programs [14]. FOAM has the potential to fill the gaps in medical training, especially in resource constrained economies [15].

Our data showed that FOAM awareness was widespread (87.5%) among doctors at the studied emergency departments. This is in keeping with the findings of Thurtle et al. in 2015 who reported that 82% of emergency medicine doctors in both developed and low resource settings were aware that FOAM existed [13]. With regards to FOAM usage, Pearson et al. reported that 41% of respondents in their study placed a “low” or “very low” importance on the use of social media in emergency medicine, but rather preferred to use social media for their own personal use [16]. This trend could be changing as evidenced by our findings whereby more than three-quarter of respondents (78.8%) indicated that they had used FOAM. The majority of study participants used FOAM on a weekly basis (57.3%), which was much better than the findings of Thurtle et al., where most participants only used FOAM 1-2 times per month [13]. Due to its longer battery life, cost effectiveness and portability, it is not surprising that most study participants (87.5%) accessed FOAM blogs using their smartphone [17].

Although females have been proven to be more proficient when it comes to the ease of use of social media, we did not find any significant differences between the proportion of females and males that used FOAM (p = 0.56) [18]. Previous studies had shown that the reluctance by some healthcare providers (HCPs) to utilize FOAM as a learning tool was attributed to its lack of peer review, possible violation of patients’ rights and information overload [19,20]. In contrast, reasons for not using FOAM among participants of this study included time constraints, lack of free Wi-Fi and unfamiliarity with social media.

There has been little study of the educational needs and time spent online by healthcare professionals in general [21]. While it is a known fact that the younger generation has readily adopted the use of technology to supplement their learning [22], increasing practitioner age (especially those older than 50 years) has been recognized as a barrier to the adoption of technologically driven health systems [23,24]. Brown et al. conducted a study on a random sample of doctors including EM doctors in Australia. They found that senior HCPs were not keen on making social media a part of their daily practice and did not subscribe to it despite the large amount of freely available information. The study further found that the extent of social media incorporation into daily practice differed greatly from individual to individual even within one hospital. The authors thought that this might be a result of limited online capabilities of senior healthcare practitioners in Australia [19]. The impact of age on the use of FOAM could not be assessed in our study as only four participants were aged older than 50 years. The lack of older doctors employed at the studied facilities may be attributed to the fact that emergency medicine is a relatively new specialty in South Africa [25]. In keeping with the findings of Burkholder et el., Life in the Fast Lane was also the most popular FOAM blog in this study [8]. EMCrit and Academic Life in Emergency Medicine have also been reported as popular emergency medicine FOAM blogs.

Low- and middle-income countries (LMIC) face many challenges in their medical training programs, one of them being a shortage of faculty staff [26]. FOAM has the potential to serve as a platform for the sharing of the latest medical information in these settings. Although EM trainees in both developed and LMIC were aware of the existence of FOAM, trainees in LMIC were less aware of specific FOAM resources [13]. Future research into FOAM usage in LMIC would be helpful to identify the gaps in educational resources and ascertain whether FOAM has the potential to mitigate this.

A limitation to our study findings is that this was a regional study that was conducted at only five hospitals in Johannesburg. Furthermore, all included facilities had an academic affiliation. Hence, our findings may not be representative of the awareness and usage of FOAM among emergency medicine doctors in general. Also, since this was a questionnaire-based study, self-reporting of data by respondents may have been subject to bias. Despite these limitations, this study has added to our understanding on the awareness and usage of FOAM in an African context. Hopefully, this study will inspire similar larger scale studies across a broader range of specialties.

## Conclusions

The level of awareness of FOAM is high and its usage prevalent among emergency medicine healthcare professionals in Johannesburg. Smartphones stood out as the preferred device utilized to access FOAM, while Life in the Fast Lane was the most popular blog. The increasing rate of adoption of FOAM by healthcare professionals is a call for the conduction of larger studies and the formal incorporation of FOAM into training curricula.
